# The Effect of 4-Methylcatechol on Platelets in Familial Hypercholesterolemic Patients Treated with Lipid Apheresis and/or Proprotein Convertase Subtilisin Kexin 9 Monoclonal Antibodies

**DOI:** 10.3390/nu15081842

**Published:** 2023-04-11

**Authors:** Lukáš Konečný, Marcel Hrubša, Jana Karlíčková, Alejandro Carazo, Lenka Javorská, Kateřina Matoušová, Lenka Kujovská Krčmová, Alena Šmahelová, Vladimír Blaha, Milan Bláha, Přemysl Mladěnka

**Affiliations:** 1 The Department of Pharmacology and Toxicology, Faculty of Pharmacy in Hradec Králové, Charles University, 50005 Hradec Králové, Czech Republic; 2 The Department of Pharmacognosy and Pharmaceutical Botany, Faculty of Pharmacy in Hradec Králové, Charles University, 50005 Hradec Králové, Czech Republic; 3 The Department of Clinical Biochemistry and Diagnostics, University Hospital Hradec Králové, 50005 Hradec Králové, Czech Republic; 4 The 3rd Department of Internal Medicine-Metabolic Care and Gerontology, University Hospital and Faculty of Medicine in Hradec Králové, Charles University, 50005 Hradec Králové, Czech Republic

**Keywords:** platelet, familial hypercholesterolemia, lipid apheresis, 4-methylcathechol

## Abstract

Elevated low-density lipoprotein (LDL) cholesterol levels lead to atherosclerosis and platelet hyperaggregability, both of which are known culprits of arterial thrombosis. Normalization of LDL cholesterol in familial hypercholesterolemia (FH) is not an easy task and frequently requires specific treatment, such as regularly performed lipid apheresis and/or novel drugs such as proprotein convertase subtilisin kexin 9 monoclonal antibodies (PCSK9Ab). Moreover, a high resistance rate to the first-line antiplatelet drug acetylsalicylic acid (ASA) stimulated research of novel antiplatelet drugs. 4-methylcatechol (4-MC), a known metabolite of several dietary flavonoids, may be a suitable candidate. The aim of this study was to analyse the antiplatelet effect of 4-MC in FH patients and to compare its impact on two FH treatment modalities via whole-blood impedance aggregometry. When compared to age-matched, generally healthy controls, the antiplatelet effect of 4-MC against collagen-induced aggregation was higher in FH patients. Apheresis itself improved the effect of 4-MC on platelet aggregation and blood from patients treated with this procedure and pretreated with 4-MC had lower platelet aggregability when compared to those solely treated with PCKS9Ab. Although this study had some inherent limitations, e.g., a low number of patients and possible impact of administered drugs, it confirmed the suitability of 4-MC as a promising antiplatelet agent and also demonstrated the effect of 4-MC in patients with a genetic metabolic disease for the first time.

## 1. Introduction

Familial hypercholesterolemia (FH) is an inherited metabolic disease characterized by elevated low-density lipoprotein cholesterol (LDL-C) levels [1]. LDL-C is the dominant risk factor for atherosclerosis, leading to coronary artery disease with an elevated cardiovascular death risk. Furthermore, it is well known that oxidized LDL-C triggers platelet aggregation in a number of ways, and the resulting hyperaggregability worsens cardiovascular events [2,3,4]. It is essential for patients to reduce LDL-C below the recommended levels by pharmacotherapy, i.e., <1.8 mmol/L, in patients with a high cardiovascular risk [5,6,7]. Lowering lipid levels in FH is challenging, and combination therapy is often required, as conventional hypolipidemic drugs in monotherapy or dual therapy (e.g., statins and/or ezetimibe) are not sufficiently active, especially in homozygous familial hypercholesterolemia (HoFH) and severe heterozygous familial hypercholesterolemia (HeFH) [5,8]. Lipid apheresis and novel proprotein convertase subtilisin kexin 9 monoclonal antibodies (PCSK9Ab) are well documented for their lipid-lowering effects [5,9]; however, their impact on platelet aggregation is barely known [4,10,11,12]. However, a recent study found that PCSK9Ab reduced the potentiating proaggregatory effect of PCSK9 on human thrombocytes ex vivo as well as in mice in vivo [4]. The effect of lipid apheresis on platelet aggregation varies from study to study [10,11,12].

Dietary intake of polyphenols has been linked to various health benefits, and polyphenols are thought to be one of the factors responsible for the health-promoting properties of the Mediterranean diet [13,14,15]. However, both human and animal feeding studies have shown that most parent polyphenolic compounds have a very low bioavailability [16]. A highly plausible explanation for this phenomenon is the extensive intestinal metabolism of polyphenols by the human microbiome. This process yields a significantly smaller spectrum of simple phenolic compounds, which possess a markedly higher bioavailability compared to their parent compounds. This hypothesis was confirmed through various feeding studies, including those exploring gnotobiotic animal models and ileostomized patients [17,18,19,20,21].

One of these metabolites, 4-methylcatechol (4-MC), has shown potent antiplatelet activity with a specific mechanism of action that could potentially circumvent the resistance frequently present to the first-line antiplatelet treatment based on acetylsalicylic acid (ASA) administration at low doses [22,23]. This metabolite is not only an efficient inhibitor of platelet aggregation, but has also shown other beneficial cardiovascular properties, such as cardioprotective and anti-hypertensive effects [24,25,26]. In addition, 4-MC also possesses neuroprotective, analgetic, antidepressant and anticancer properties [27,28,29,30,31].

Therefore, the aim of this cross-sectional study was to ascertain the antiplatelet effect of 4-MC in patients suffering from FH in general, compare the differences in platelet aggregation between different FH treatment modalities (lipid apheresis, pharmacotherapy) and perform a comparison to age-matched, generally healthy donors.

## 2. Materials and Methods

### 2.1. Donors

All 15 patients suffering from FH and coming from all parts of Czechia and treated at the University Hospital in Hradec Králové were enrolled in this study (Table 1 and Table 2). The inclusion criteria were a diagnosis of FH, insufficient response to standard hypolipidemic therapy and the presence of atherosclerosis; for the apheresis group, criteria also included long-term apheresis treatment. The exclusion criteria were other severe and acute illnesses (chronic inflammatory diseases, acute infections and autoimmune or malignant disorders). Patients were treated according to the current guidelines [5,8,32,33]. A novel treatment with PCSK9Ab was used in 12 out of 15 FH patients, and 8 of 15 FH patients were undergoing lipid apheresis. For the purpose of this study and to facilitate a reasonable comparison, patients were divided into 2 groups according to treatment modality (Table 3 and Table 4): (1) the apheresis group, with 6 patients treated with PCSK9Ab and undergoing lipid apheresis at the same time, and (2) the pharmacotherapy group with 6 patients who were treated with PCSK9Ab. The remaining 3 patients, who were neither included in the apheresis group nor the pharmacotherapy group, as none of them was treated with PCKS9Ab (Table 2), were grouped together with the abovementioned 12 patients (6 in each group, pharmacotherapy and apheresis) in the analysis comparing FH patients vs. age matched generally healthy controls (15 vs. 15 cases). The latter control group was selected from our previous research, where the inclusion criteria in generally healthy donors were age ≥ 18 years and subjectively reported good health condition. The exclusion criteria were the presence of any cardiovascular disease or risk factors for atherosclerosis and the use of drugs that affect platelet function. Details were previously reported [34]. All donors signed an informed consent in line with the approval of ethics committee of the University Hospital in Hradec Králové (No. 202007 S01P from 18 June 2020). All experiments conformed to the latest Declaration of Helsinki. 

### 2.2. Blood and Urine Collection

Drugs known to influence platelet aggregation, including non-steroidal anti-inflammatory drugs, and alcohol were not allowed 24 h prior to blood draw. In some FH patients, antiplatelet drugs were administered daily as they were indicated due to presence of a cardiovascular disease and could not be discontinued for ethical reasons (Table 2). Blood samples for aggregation experiments were collected into tubes containing heparin sodium (17 IU/mL). Blood draws were always performed in the morning (at 8–9 a.m.). In the apheresis group, post-apheresis samples were also taken after the end of the procedure, which was approximately 4 h after the pre-apheresis samples were taken. A small volume of morning urine sample was collected from all donors.

### 2.3. Lipid Apheresis

Lipid apheresis was carried out from the peripheral vein in the elbow pit or in the forearm. Plasma separation was performed using a Cobe-Spectra or Optia continuous centrifugal separator (Terumo, Likewood, CO, USA), and washed plasma with erythrocytes was returned to another peripheral vein. The procedure resulted in purification of 100–150% of the circulating plasma volume. A mixture of heparin and dextrose citrate solution A (Baxter, Munich, Germany) was used as an anticoagulant. Three different apheresis principles were used. An immunoadsorption Lipopak adsorber column (Pocard, Moscow, Russia) employing sheep antibodies against apolipoprotein B 100 was used in three patients. In 2 other patients, a Lipocollect column (Medicollect, Ullrichstein, Germany) was used. In the remaining 3 FH patients, due to the co-presence of hyperfibrinogenaemia, rheohaemapheresis was used according to Prof. Borberg et al. with our own modification [35,36] by employing Evaflux 4A filter (Kawasumi, Tokyo, Japan). The flow through the filter was controlled using the CF100 automatic machine (Infomed, Geneva, Switzerland). 

### 2.4. Chemicals

Phosphate buffer, dimethyl sulphoxide (DMSO), ASA, ticagrelor, ristomycin monosulphate (ristocetin), platelet-activating factor-16 (PAF) and 9,11-dideoxy-11α,9α-epoxymethanoprostaglandin F_2α_ (U-46619) were purchased from Sigma-Aldrich (St. Louis, MO, USA). Thrombin receptor agonist peptide-6 (TRAP), adenosine-5-diphosphate (ADP) and arachidonic acid (AA) were purchased from Roche (Basel, Switzerland). Vorapaxar was purchased from Selleck Chemicals GmbH (Planegg, Germany). Collagen was purchased from Diagnostica, a.s. (Prague, Czechia) while 0.9% sodium chloride (saline) was purchased from B. Braun (Melsungen, Germany).

### 2.5. Platelet Aggregation Experiments

A total of 300 µL of whole blood was diluted with the same volume of preheated 0.9% NaCl solution and incubated with 5 µL of 4-MC or with 5 µL of the used solvent (DMSO) at a final concentration of 0.8% for 3 min at 37 °C. Platelet aggregation was triggered by AA, collagen or ristocetin (inductors) and monitored for 6 min. The aggregation response was quantified using the AUC (area under the curve). The final concentrations of the inducers and inhibitor are given in Table 5.

Due to relatively large volume of collected blood (samples taken pre- and post-apheresis) in FH patients undergoing lipid apheresis, some analyses were not performed (collagen triggered reaction after preincubation with higher concentration of 4-MC/70 µM/and aggregation induced by ristocetin after pretreatment with 4-MC) in contrast to FH patients treated solely via pharmacotherapy.

### 2.6. Measurement of Biochemical Parameters

Glucose, total cholesterol (TC), HDL cholesterol (HDL-C), LDL-C and triglycerides (TGs) were measured in serum using commercial enzymatic kits by the Cobas 8000 system (Roche, Basel, Switzerland). Creatinine was determined in both serum and urine. Analysis was carried out using the Prominence LC 20 HPLC set with the SPD-M20A Shimadzu (Shimadzu, Kyoto, Japan) diode array detector. As the stationary phase, two monolithic columns RP-18e (4.6 mm × 50 mm, 3.0 mm × 100 mm) were connected together in combination with a 15 mM phosphate buffer as the mobile phase. Creatinine was detected at 235 nm using diode array detection [37].

### 2.7. Statistical Analysis

GraphPad Prism 9.3.1. (GraphPad Software, San Diego, CA, USA) was used for all data analyses. Firstly, data were tested using the Shapiro–Wilk test for confirming or rejecting the Gaussian distribution. Based on this analysis, either parametric or non-parametric tests were used. In the former case, a Student’s *t*-test or paired *t*-test was used, while in the latter case, Mann–Whitney or Wilcoxon matched-pairs signed-rank tests were employed. Categorical parameters were analysed using the chi-square test.

## 3. Results and Discussion

**Figure 1:**
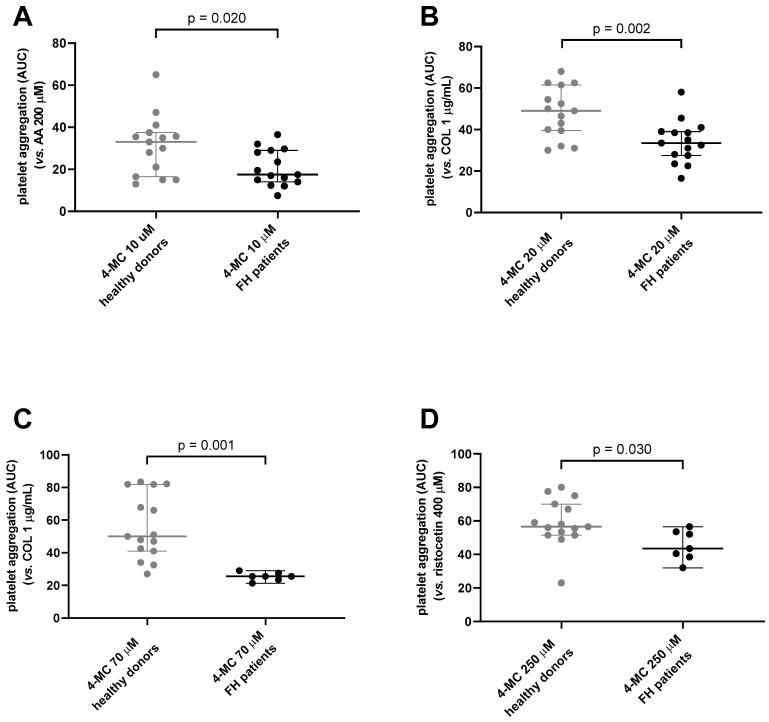
Comparison of the effect of 4-methylcatechol (4-MC) on platelet aggregation between a group of healthy donors and familial hypercholesterolemic (FH) patients. Results are shown as area under the curve (AUC) of platelet aggregatory responses. (**A**) Platelet aggregation induced by arachidonic acid (AA) in blood pretreated with 10 µM 4-MC. (**B**) Platelet aggregation induced by collagen (COL) in blood pretreated with 20 µM 4-MC. (**C**) Platelet aggregation induced by collagen in blood pretreated with 70 µM 4-MC. (**D**) Platelet of aggregation induced by ristocetin in blood pretreated with 250 µM 4-MC. Pictures (**A**,**B**) include all 15 FH patients compared to 15 age-matched, generally healthy controls. Pictures (**C**,**D**) include 7 FH patients treated with pharmacotherapy (without apheresis) compared to 15 age-matched controls. The effect of ristocetin and higher concentrations of 4-MC was tested solely in patients that were not treated with apheresis in order to reduce the volume of drawn blood before and after the unpleasant procedure. Results are shown as median with 95% confidence interval.

**Figure 2:**
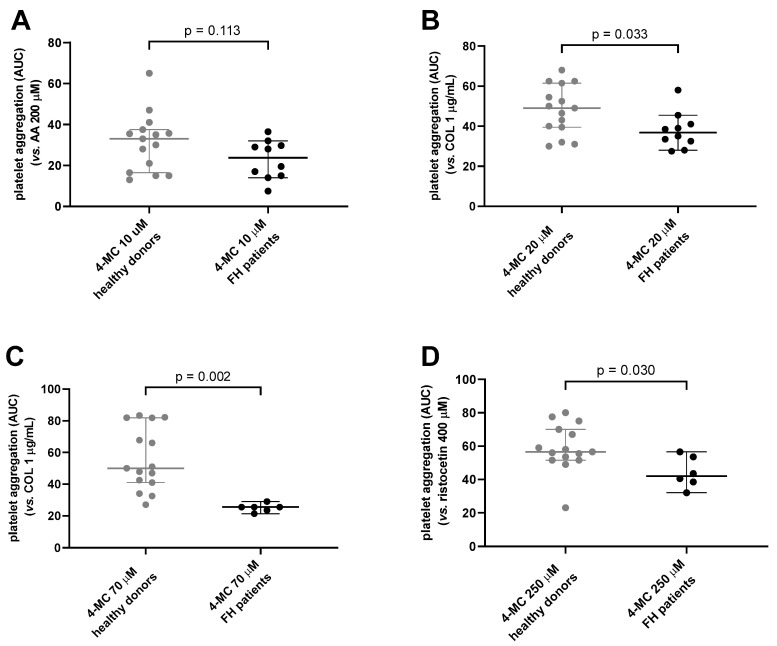
Comparison of the effect of 4-methylcatechol (4-MC) on platelet aggregation between a group of generally healthy donors and familial hypercholesterolemic (FH) patients after exclusion of FH patients treated with conventional antiplatelet drugs. Results are shown as area under the curve (AUC) of platelet aggregatory responses. (**A**) Platelet aggregation induced by arachidonic acid (AA) in blood pretreated with 10 µM (4-MC). (**B**) Platelet aggregation induced by collagen (COL) in blood pretreated with 20 µM 4-MC. (**C**) Platelet aggregation induced by collagen in blood pretreated with 70 µM 4-MC. (**D**) Platelet of aggregation induced by ristocetin in blood pretreated with 250 µM 4-MC. Pictures (**A**,**B**) encompass 10 FH patients that did not undergo conventional antiplatelet therapy (2 patients with enteric-coated ASA were included) compared to 15 age-matched, generally healthy controls. Pictures (**C**,**D**) include 6 FH patients treated solely pharmacologically (without apheresis) again without conventional antiplatelet therapy compared to 15 age-matched, generally healthy controls. The effect of ristocetin and higher concentrations of 4-MC was tested only in patients that were not treated with apheresis in order to reduce the volume of drawn blood before and after the unpleasant procedure. Results are shown as median with 95% confidence interval.

In our previous study with healthy controls [23], we showed that 4-MC is an active antiplatelet drug with higher potency than the first-line antiplatelet drug, ASA. Hence, as the first step of this study, the antiplatelet effect of 4-MC in 15 FH patients was compared with its effect on 15 age-matched, generally healthy donors selected from our previous study in order to assess if 4-MC is also active in patients suffering from FH. There were no differences between these groups in basic characteristics with the exception of the number of smokers, cholesterol levels and indicated antiplatelet drugs (Table 2). Cholesterol levels as well as the number of smokers were higher in the control group, while antiplatelet drugs were used by 7 of 15 FH patients. Notwithstanding the limitation caused by the impossibility of discontinuing the current antiplatelet treatment in those patients, the antiplatelet potential of 4-MC was also confirmed in FH patients (Figure 1), which suggested that 4-MC is active despite the use of ASA or its combination with clopidogrel. Moreover, 4-MC had a stronger antiplatelet effect in this group of FH patients compared to age-matched controls. This was observed after AA, collagen and ristocetin triggered platelet aggregation (Figure 1). This means that in general, the antiplatelet effect of 4-MC was stronger in our well-treated FH patients than in an age-matched healthy control group. As concomitant antiplatelet therapy may logically bias the results, the analysis was repeated solely with FH patients who were not taking antiplatelet therapy. When these seven patients were excluded, solely the higher concentration of 4-MC remained significantly more active in FH patients than in controls after collagen-triggered aggregation (*p* = 0.003), and there was a tendency in ristocetin-induced aggregation for 4-MC to be more active in FH patients (*p* = 0.06). As two patients were taking enteric-coated ASA, which is very probably not active as an antiplatelet treatment because ASA is rapidly metabolized into salicylic acid in the intestine in contrast to conventional ASA, which is absorbed in the stomach and blocks platelet activity immediately in the portal vein [38,39,40], we considered these patients as not taking antiplatelet treatment for our future analysis. This analysis confirmed the results obtained with all 15 FH patients. The effect of 4-MC in collagen- and ristocetin-induced aggregation was higher in FH patients than in controls. Solely the difference in AA-triggered aggregation was not significant after this adjustment (Figure 2). This was quite logical as the five excluded patients were taking conventional ASA, which blocks transformation of AA into prostaglandin H_2_. There are probably two reasons that contributed to the differences in the antiplatelet effect between FH patients and controls: (1) 4-MC improved the effect of basal antiplatelet treatment in these patients, and indeed, our previous study suggested an additive effect of 4-MC on ASA [23]; (2) decreased plasma cholesterol is associated with lower platelet aggregation [41,42], and this was likely even potentiated by 4-MC.

In addition, we also assessed if smoking, which was more prevalent in controls, could not also be partly responsible for the difference. Pre-analysis of all tested donors (FH + controls) and smokers vs. non-smokers suggested such a difference at least in collagen-induced aggregation with 4-MC premedication (Supplementary Data Table S1). Hence, in our next subanalysis, we excluded all tobacco smokers. This exclusion, however, did not substantially modify the results: 4-MC remained more active in FH patients than in the control group after platelet aggregation was triggered by all three used inducers ( Supplementary Data Figure S1).

In the next step, the effects of different treatment modalities were compared, i.e., (1) the impact of lipid apheresis and (2) comparison between the combination of lipid apheresis with PCKS9Ab pharmacotherapy and the latter approach. There were no differences between these groups in major characteristics, including administration of antiplatelet drugs (Table 3), but significant changes were observed in lipid levels (Table 4). There are some previous data suggesting that lipid apheresis decreases platelet aggregation via several inducers. Pares et al. showed a reduction in ADP-induced platelet aggregation immediately after apheresis [12], while some other studies suggested the benefit of apheresis on the same platelet inductor ADP only after several weeks, presumably as a result of prolonged platelet survival [10]. Lower platelet survival occurs in atherosclerotic illnesses [43], and it can be assumed that elimination of LDL-C leads to gradual normalization of atherosclerosis and prolongation of platelet survival. However, almost no benefit of lipid apheresis was observed after ADP or ultrasound-induced aggregation in another study [11].

**Figure 3:**
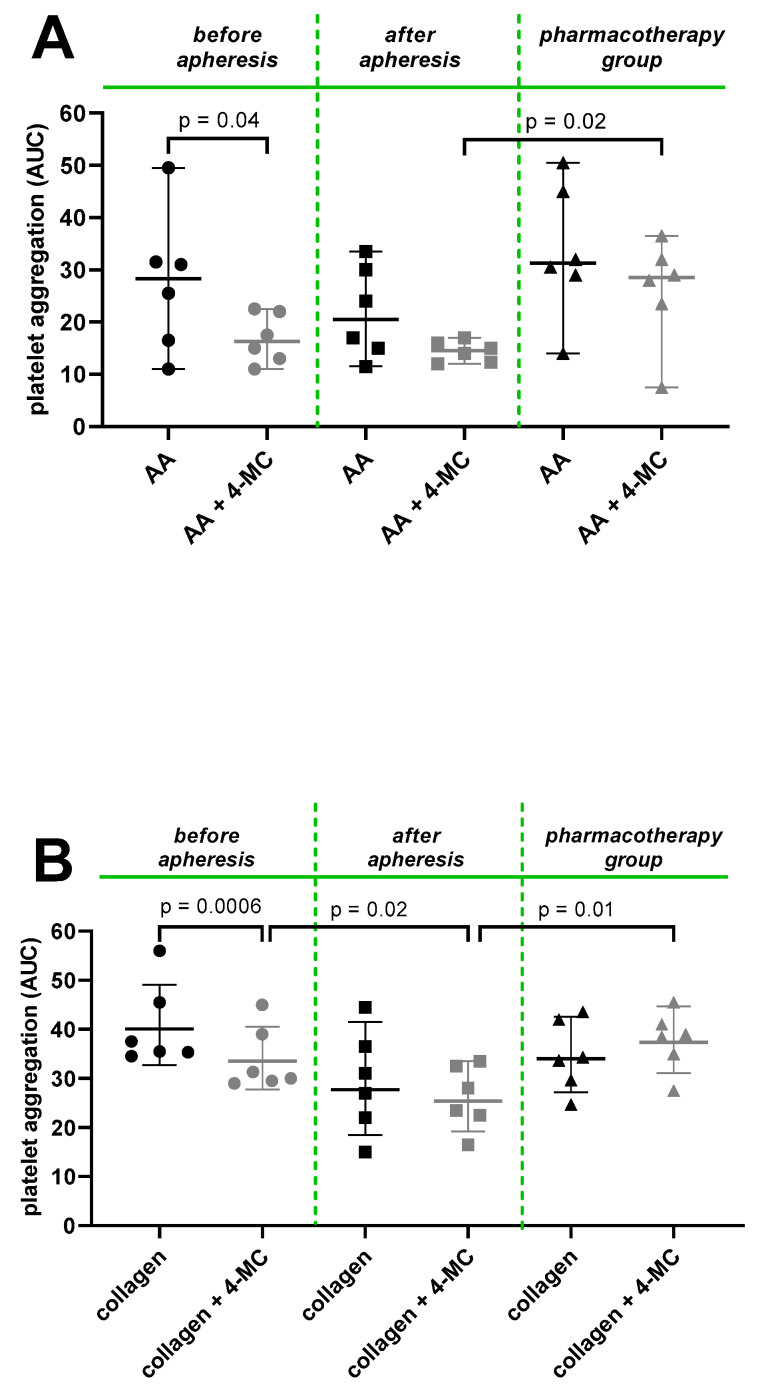
The effect of lipid apheresis and comparison between apheresis group and pharmacotherapy group on the antiplatelet activity of 4-methylcatechol (4-MC). The apheresis group included 6 patients treated with lipid apheresis + monoclonal antibodies against proprotein convertase subtilisin kexin 9 (PCSK9Ab) + ezetimibe. Some patients also received a statin and lomitapide, while the 6 patients in the pharmacotherapy group were not treated with any kind of lipid apheresis but were chronically administered PCKS9Ab, a statin and ezetimibe. Results are shown as area under the curve (AUC) of platelet aggregatory responses. (**A**) Platelet aggregation induced by arachidonic acid (AA) at a final concentration of 200 µM with or without pretreatment with 4-MC at a final concentration of 10 µM. (**B**) Platelet aggregation induced by collagen at a final concentration of 1 µg/mL with or without pretreatment with 4-MC at final concentration of 20 µM. *p*-values were calculated using corresponding paired (pre- and post-apheresis) or an unpaired statistical tests (apheresis samples vs. pharmacotherapy group). Results are shown as median with 95% confidence interval.

In our study with six FH patients treated with lipid apheresis, this procedure had no significant impact on AA- or collagen-based aggregation but partly improved the antiplatelet effect of 4-MC (Figure 3—before and after apheresis data). The improved effect of 4-MC was observed after collagen-induced platelet aggregation. In the case of AA-triggered aggregation, the results before and after apheresis were essentially similar (Figure 3), which means that lipid apheresis did not improve the effect of 4-MC on AA-triggered aggregation.

There were no differences in AA- and collagen-induced platelet aggregation between the six patients in the apheresis group before the procedure of apheresis and the six patients in the pharmacotherapy group. This is not very surprising as both groups were treated with PCKS9Ab. However, the augmenting effect of lipid apheresis can also be seen here, as after the lipid apheresis platelet aggregation responses to both AA and collagen in 4-MC-pretreated samples were significantly lower when compared to the pharmacotherapy group (Figure 3A,B). Hence, lipid apheresis always improved the antiplatelet effect of 4-MC on collagen, but the effect on AA-triggered aggregation improved only when apheresis and pharmacotherapy groups were compared. The significance of this latter finding is unknown so far, but it should be stated that collagen is one of the most relevant inducers of platelet aggregation as damage to vessels reveals subendothelial collagen layers, and this propagates the process of thrombus formation [44]. Based on measured LDL cholesterol levels (Table 4) before and after apheresis and in the pharmacotherapy group, it seems that cholesterol level was the major determinant of the magnitude of 4-MC antiplatelet effect as the strongest effect was observed in blood samples after apheresis. This suggests that the type of treatment modality is not a crucial factor in contrast to the LDL cholesterol levels. Similar findings were also observed previously; for example, there is a highly linear relationship between serum cholesterol and formation of proaggregatory mediator thromboxane A_2_ in washed platelets stimulated by AA [42], and patients with lower total cholesterol levels have a better response to ASA than those with higher levels [41].

## 4. Study Limitations

The authors of this study fully acknowledge that due to the low number of patients included, the outcomes should be considered indicative, and no definitive conclusions can be drawn from such a restricted patient sample. On the other hand, the genetic disease we studied is rare and obtaining patients meeting the inclusion criteria is exceptionally difficult as both PCKS9Ab and lipid apheresis are highly expensive procedures limited to the indicated patients. There are no more patients in Czechia that can be included. Moreover, working with platelets requires a very tightly defined schedule in order to obtain reproducible data, and this is best accomplished in one centre. Another crucial limitation was the inability to exclude FH patients treated with antiplatelet drugs. Given the inclusion criteria encompassing atherosclerosis, it was not possible to discontinue these drugs. This limitation was not an issue in the comparison of apheresis vs. pharmacotherapy in FH patients, but it was an important drawback when comparing FH patients vs. healthy persons. Moreover, enteric-coated ASA likely has no or limited antiplatelet profit [38,39,40], and this further complicated the analysis. We performed subsequent analyses by excluding those patients, but the sensitivity of these analyses was lower due to the low number of remaining cases.

## 5. Conclusions

In this study, a promising antiplatelet effect of 4-MC, which has already been seen with healthy volunteers, was confirmed. Importantly, 4-MC also showed its antiplatelet effects in patients with a genetic metabolic disease for the first time. Our analyses seem to support the hypothesis that 4-MC has more significant antiplatelet effects in well-managed FH patients than generally healthy controls, and we suppose that the major reason for this is simply the lower lipid levels in FH patients. However, given the inherent limitation of this study, further investigation with an expanded population sample is needed to reach a definitive conclusion.

## Figures and Tables

**Table 1:** Basic clinical and genetic characterisation of recruited patients.

	Patient No. (Sex)	Therapy at the Time of Analysis	Phenotype	Gene	Exone Location No.
pharmacotherapy group	1 (M)	**PCSK9Ab** + S + E	HeFH	UN	E4–E8
	2 (M)	**PCSK9Ab** + S + E	HeFH	LDL-R	E9
	3 (F)	**PCSK9Ab** + S + E	HeFH	LDL-R	E8
	4 (M)	**PCSK9Ab** + S + E	HeFH	LDL-R	E7
	5 (M)	**PCSK9Ab** + S + E	HeFH	LDL-R	UN
	6 (M)	**PCSK9Ab** + S + E	HeFH	LDL-R	UN
	7 (M)	S + E	HeFH	LDL-R	E12
apheresis group	8 (F)	**Lipid apheresis** + **PCSK9Ab** + S + E	HoFH	LDL-R	E8
	9 (F)	**Lipid apheresis** + **PCSK9Ab** + S + E	HeFH	UN	UN
	10 (M)	**Lipid apheresis** + **PCSK9Ab** + S + E	HeFH	UN	E9–E14
	11 (F)	Lipid apheresis + S + E	HeFH	UN	UN
	12 (M)	Lipid apheresis + S + Fi	HeFH	UN	UN
	13 (M)	**Lipid apheresis** + **PCSK9Ab** + S + E + L	HoFH	LDL-R	E12; E16
	14 (F)	**Lipid apheresis** + **PCSK9Ab** + S + E	HoFH	LDL-R	UN
	15 (F)	**Lipid apheresis** + **PCSK9Ab** + S + E + L	HoFH	LDL-R	E10; E12

F: female; HeFH: heterozygous familial hypercholesterolemia; HoFH: homozygous familial hypercholesterolemia; LDL-R: low-density lipoprotein receptor; M: male; No.: number; UN: unknown. Following drugs according to ATC code S: statins (C10AA); Fi: fenofibrate (C10AB); E: ezetimibe (C10AX09); L: lomitapide (C10AX12); PCSK9Ab (C10AX13-14). Patients included in the analysis of pharmacotherapy and apheresis groups are highlighted in bold.

**Table 2:** Comparison of healthy group and FH patients.

		Healthy Donors	FH Patients	*p*-Value
age	40–77	59 ± 6	52 ± 11	0.127
BMI	18.5–30+	29.27 ± 4.49	26.42 ± 3.29	0.128
smokers—N (%)	Yes	6 (40%)	1 (7%)	**0.031**
COVID-19 ^a^—N (%)	Yes	7 (47%)	5 (33%)	0.456
enteric-coated ASA—N (%)	Yes	0 (0%)	2 (13%)	0.143
conventional ASA—N (%)	Yes	0 (0%)	3 (20%)	0.068
clopidogrel + ASA combination—N (%)	Yes	0 (0%)	2 (13%)	0.143
biochemical parameters	TC (mmol/L)	5.53 ± 0.78	4.30 ± 1.48	**0.010**
	LDL-C (mmol/L)	3.46 ± 0.73	2.52 ± 1.38	**0.006**
	glucose (mmol/L)	5.45 ± 0.58	6.10 ± 2.05	0.336
	HDL-C (mmol/L)	1.52 ± 0.43	1.18 ± 0.33	**0.027**
	TG (mmol/L)	1.37 ± 0.45	1.81 ± 1.12	0.250
	creatinine in serum (µmol/L)	80.54 ± 12.77	76.37 ± 17.22	0.314
	creatinine in urine (mmol/L)	9.99 ± 4.17	10.86 ± 6.93	0.921

ASA: acetylsalicylic acid; BMI: body mass index; FH: familial hypercholesterolemia; HDL-C: HDL cholesterol; LDL-C: LDL cholesterol; N: number of patients; TC: total cholesterol; TGs: triglycerides. Data are shown as mean ± SD. Per cent values are related to the total number of subjects in the healthy donor group (*n* = 15) or FH patient group (*n* = 15). *p*-values were calculated using an unpaired *t*-test or chi-square test. Body mass index was calculated with the known formula: weight/(height [m])^2^. ^a^ diagnosed with COVID-19 at different time points between 3 and 7 months prior to blood withdrawal.

**Table 3:** Basic characterisation of apheresis and pharmacotherapy groups.

		Apheresis Group	Pharmacotherapy Group	*p*-Value
age	40–77	49 ± 13	51 ± 6	0.585
BMI	18.5–30+	28.00 ± 4.72	32.32 ± 4.08	0.138
smokers—N (%)	Yes	0 (0%)	0 (0%)	-
COVID-19 ^a^—N (%)	Yes	2 (33%)	3 (50%)	0.558
enteric-coated ASA—N (%)	Yes	1 (16%)	1 (16%)	0.999
conventional ASA—N (%)	Yes	2 (33%)	1 (16%)	0.505
clopidogrel + ASA combination—N (%)	Yes	1 (16%)	0 (0%)	0.296
cardiovascular diseases—N (%)	arterial hypertension	2 (33%)	0 (0%)	0.121
	ACB	1 (16%)	2 (33%)	0.505
	AS of carotid arteries	2 (33%)	2 (33%)	0.999
	CAD	3 (50%)	3 (50%)	0.999
	PAD	1 (16%)	0 (0%)	0.296
	AS and calcifications/AS and defect/just AS of aortic valve	3 (50%)	1 (16%)	0.221
	stroke	1 (16%)	0 (0%)	0.296
	moderate stenosis of left ACC	1 (16%)	0 (0%)	0.296
	bilateral AS and calcifications of ACC	1 (16%)	0 (0%)	0.296
	haemodynamically non-significant AO stenosis	1 (16%)	0 (0%)	0.296
other diseases—N (%)	familial hypercholesterolemia	6 (100%)	6 (100%)	
	diabetes mellitus type 1	1 (16%)	1 (16%)	0.999
	hypothyreosis	3 (50%)	0 (0%)	**0.045**
	anaemia	1 (16%)	0 (0%)	0.296
	allergy	1 (16%)	0 (0%)	0.296
	glaucoma	1 (16%)	0 (0%)	0.296
	asthma	1 (16%)	0 (0%)	0.296

ACB: aortocoronary bypass; ACC: arteria carotis communis; AO: aortic; AS: atherosclerosis; ASA: acetylsalicylic acid; BMI: body mass index; CAD: coronary artery disease; CVD: cardiovascular disease; N: number of patients; PAD peripheral artery disease. Data are shown as mean ± SD. Per cent values are related to the total number of subjects in the group undergoing apheresis (*n* = 6) and in pharmacotherapy (without-apheresis) group (*n* = 6). *p*-values were calculated using an unpair *t*-test or chi-square test. Body mass index was calculated with the known formula: weight/(height [m])^2^. ^a^ diagnosed with COVID-19 at different time points between 3 and 7 months prior to blood withdrawal.

**Table 4:** Biochemical characterisation of apheresis and pharmacotherapy groups.

Patients, N (%)		Apheresis Group	Pharmacotherapy Group	*p*-Value
biochemical parameters	TC (mmol/L)	^A ^4.73 ± 1.93 ^B^ 1.82 ± 0.55	^C ^3.81 ± 1.12	**A vs. B 0.010** A vs. C 0.378 **B vs. C 0.005**
	LDL-C (mmol/L)	^A ^2.92 ± 1.89 ^B^ 0.67 ± 0.49	^C ^2.22 ± 0.93	**A vs. B 0.019** A vs. C 0.479 **B vs. C 0.008**
	glucose (mmol/L)	^A ^5.52 ± 0.85 ^B^ 6.58 ± 1.84	^C^ 6.03 ± 0.98	A vs. B 0.215 A vs. C 0.394 B vs. C 0.585
	HDL-C (mmol/L)	^A ^1.34 ± 0.38 ^B^ 1.03 ± 0.30	^C^ 1.08 ± 0.20	**A vs. B 0.005** A vs. C 0.155 B vs. C 0.944
	TG (mmol/L)	^A ^1.36 ± 0.76 ^B^ 0.65 ± 0.39	^C^ 1.52 ± 0.49	**A vs. B 0.011** A vs. C 0.707 **B vs. C 0.011**
	creatinine in serum (µmol/L)	^A ^66.16 ± 32.82 ^B^ 64.26 ± 30.77	^C^ 77.14 ± 8.69	A vs. B 0.434 A vs. C 0.880 B vs. C 0.687
	creatinine in urine (mmol/L)	^A ^7.35 ± 5.81	^C^ 14.98 ± 6.80	A vs. C 0.085

N: number of patients; TC: total cholesterol; HDL-C: HDL cholesterol, LDL-C: LDL cholesterol; TGs: triglycerides. Data are shown as mean ± SD. Data are related to subjects in the groups undergoing apheresis (*n* = 6) and pharmacotherapy (without-apheresis) group (*n* = 6). *p*-values were calculated using a paired *t*-test (^A^ before and ^B^ after apheresis) or an unpaired *t*-test (^A^ or ^B^ apheresis samples vs. ^C^ pharmacotherapy group). Creatinine in urine was measured only before the apheresis procedure.

**Table 5:** Final concentrations of inducers and 4-MC.

		Final Concentration	Units
inducers	collagen	1	µg/mL
	AA	200	µM
	ristocetin	400	µM
inhibitor	4-MC	10 ^A^, 20 ^C^, 70 ^C^ and 250 ^R^	µM

^A^: for AA experiments, ^C^: for collagen experiments, ^R^: for ristocetin experiments.

## Data Availability

Raw data from this study are related to real patients and hence cannot be publicly shared, but they are available upon a justified request to the corresponding author.

## Supplementary Materials

The following supporting information can be downloaded at https://www.mdpi.com/article/10.3390/nu15081842/s1: Table S1. Statistical comparison between all smokers and all non-smokers. Figure S1. Comparison of the effect of 4-methylcatechol (4-MC) on platelet aggregation between a group of generally healthy donors and familial hypercholesterolemic patients, where smokers were excluded from both groups. Results are shown as area under the curve (AUC) of platelet aggregatory responses.
