# Supplementary material for: The Effect of 4-Methylcatechol on Platelets in Familial Hypercholesterolemic Patients Treated with Lipid Apheresis and/or Proprotein Convertase Subtilisin Kexin 9 Monoclonal Antibodies
Source: Nutrients. 2023 Apr 11;15(8):1842. doi: 10.3390/nu15081842 (PMC10143685; doi:10.3390/nu15081842)
Supplement: Supplementary file 1 [file nutrients-15-01842-s001.zip › nutrients-2280469-supplementary.docx]

The effect of 4-methylcatechol on platelets in familial hypercholesterolemic patients treated with lipid apheresis and/or proprotein convertase subtilisin kexin 9 monoclonal antibodies

Lukáš Konečný ^1^, Marcel Hrubša ^1^, Jana Karlíčková^2^, Alejandro Carazo^1^, Lenka Javorská^3^, Kateřina Matoušová^3^, Lenka Kujovská Krčmová^3^, Alena Šmahelová^4^, Vladimír Blaha^4^, Milan Bláha^4^ and Přemysl Mladěnka^1, *^

|  |
| --- |

^1^ The Department of Pharmacology and Toxicology, Faculty of Pharmacy in Hradec Králové, Charles University, 50005 Hradec Králové, Czechia

^2^ The Department of Pharmacognosy and Pharmaceutical Botany, Faculty of Pharmacy in Hradec Králové, Charles University, 50005 Hradec Králové, Czechia

^3^ The Department of Clinical Biochemistry and Diagnostics, University Hospital Hradec Králové, 50005 Hradec Králové, Czechia

^4^ The 3rd Department of Internal Medicine-Metabolic Care and Gerontology, University Hospital and Faculty of Medicine in Hradec Králové, Charles University, 50005 Hradec Králové, Czechia

***** Correspondence: mladenkap@faf.cuni.cz; Tel.: +420-495-067-295

SUPPLEMENTARY DATA

**3 pages**

Table S1. Statistical comparison between all smokers and all non-smokers (patients with familial hypercholesterolemia and controls were analysed together).

| inductor | inhibitor | Patients, N (%) | smokers | non-smokers | p-value |
| --- | --- | --- | --- | --- | --- |
| AA | 4-MC 10 µM |  | 7 (23 %) | 23 (77 %) | 0.422 |
| collagen | 4-MC 20 µM |  | 7 (23 %) | 23 (77 %) | **0.036^b^** |
| collagen^a^ | 4-MC 70 µM |  | 4 (13 %) | 10 (33 %) | 0.134 |
| ristocetin^a^ | 4-MC 250 µM |  | 4 (13 %) | 10 (33 %) | 0.205 |

4-MC: 4-methylcathechol; AA: arachidonic acid; FH: familial hypercholesterolemia; N: number of patients.

Per cent values are related to the total number of healthy donor and FH patient (n=30). P-values were calculated by a parametric or non-parametric unpair t-test.

^a^The effect of ristocetin and higher concentration of 4-MC was tested solely in patients non-treated with apheresis in order to reduce the volume of drawn blood before and after the patient’s unpleasant procedure.

^b^The effect of 4-MC was lower in smokers.

**Figure S1.** Comparison of the effect of 4-methylcatechol (4-MC) on platelet aggregation between a group of generally healthy donors and familial hypercholesterolemic (FH) patients, where smokers were excluded from both groups. Results are shown as area under the curve (AUC) of platelet aggregatory responses. **A:** Platelet aggregation induced by arachidonic acid (AA) in blood pre-treated with 10 µM (4-MC). **B:** Platelet aggregation induced by collagen (COL) in blood pre-treated with 20 µM 4-MC. **C:** Platelet aggregation induced by collagen in blood pre-treated with 70 µM 4-MC. **D:** Platelet of aggregation induced by ristocetin in blood pre-treated with 250 µM 4-MC. Pictures **A** and **B** included 14 non-smokers FH patients compared to 9 non-smokers from age-matched generally healthy control group. Pictures **C** and **D** included 7 non-smokers FH patients treated for pharmacotherapy (without apheresis) compared to 9 non-smokers from age-matched generally healthy control group. The effect of ristocetin and higher concentration of 4-MC was tested solely in patients non-treated with apheresis in order to reduce the volume of drawn blood before and after the patient’s unpleasant procedure. Results are shown as median with 95% confidence interval.
